# A structural comparison of lipopolysaccharide biosynthesis loci of *Legionella pneumophila* serogroup 1 strains

**DOI:** 10.1186/1471-2180-13-198

**Published:** 2013-09-04

**Authors:** Markus Petzold, Alexander Thürmer, Susan Menzel, Johan W Mouton, Klaus Heuner, Christian Lück

**Affiliations:** 1 Institute of Medical Microbiology and Hygiene, Faculty of Medicine ‘Carl Gustav Carus’, University of Technology Dresden, Fetscherstraße 74, Dresden D-01307, Germany; 2 Department of Medical Microbiology, Radboud University Nijmegen Medical Centre, PO Box 9101, Nijmegen, Netherlands; 3 Cellular Interactions of Bacterial Pathogens, Centre for Biological Security, Division 2 (ZBS2), Robert Koch-Institute, Nordufer 20, Berlin D-13353, Germany

**Keywords:** *Legionella pneumophila*, Lipopolysaccharide, Locus organization, Monoclonal antibody typing

## Abstract

**Background:**

The lipopolysaccharide (LPS) is the major immuno-dominant antigen of all *Legionella* species including *L. pneumophila*. Its diversity is the basis for the classification of *L. pneumophila* into serogroups and monoclonal subgroups and is thought to be involved in strain specific virulence. The understanding of the genetic basis of the LPS-antigen is incomplete. Thus, we analyzed the genetic locus involved in LPS-biosynthesis of *L. pneumophila* serogroup 1 (Sg1) strains with the focus on strain specific gene composition.

**Results:**

The LPS-biosynthesis loci of 14 *L. pneumophila* Sg1 strains comprise two distinct regions: A 15 kb region containing LPS-biosynthesis genes that can be found in all *L. pneumophila* strains and a Sg1-specific 18 kb region. The 15 kb region is highly conserved among Sg1 strains as reflected by high homologies of single ORFs and by a consistent ORF arrangement. In contrast, the Sg1 specific 18 kb region is variable and partially disrupted by phage related genes. We propose that the region spanning from ORF 6 to ORF 11 of the Sg1-specific region is likely involved in late LPS-modification. Due to the high variability of this small region and various combinations of single ORFs within this region a strain specific LPS-structure could be synthesized including modifications of legionaminic acid derivates.

**Conclusions:**

Our data clearly demonstrate that the gene structure of the LPS-biosynthesis locus of *L. pneumophila* Sg1 strains show significant interstrain variability. These data can be used for further functional analysis of the LPS synthesis to understand pathogenesis and reactivity with monoclonal antibodies. Moreover, variable but strain specific regions can serve as basis for the development of novel genotyping assays.

## Background

*Legionella pneumophila* is one of 56 described species belonging to the genus *Legionella* of the family Legionellaceae [1]. These Gram-negative bacteria are ubiquitous inhabitants of natural and manmade aquatic environments where they survive parasitically in protozoa like amoeba [2,3] and in community structures such as biofilms [4,5]. Additionally, *Legionella* can infiltrate the human lung via inhaled aerosols [3,6] and subsequently infect alveolar macrophages [7] which frequently cause a potential fatal pneumonia termed Legionnaires’ disease (LD) [8]. *L. pneumophila* strains belonging to the serogroup 1 (Sg1) were predominantly reported in LD cases, especially in community acquired and travel-associated cases [9,10].

Lipopolysaccharide (LPS) is the major immuno-dominant antigen of all *Legionella* species including *L. pneumophila*[11]. It is the main component recognized by patient’s sera and by diagnostic assays in urinary antigen detection [12]. The LPS molecule possesses a high degree of diversity and thereby provides the basis for the classification of *L. pneumophila* into serogroups and subgroups by monoclonal antibodies (mAb) [13-15]. Sg1 strains are subdivided into nine mAb-subgroups using the Dresden monoclonal antibody panel (Table 1) [16].

**Table 1:** **Monoclonal antibody based subgrouping of *L. pneumophila *Sg1 strains using the Dresden panel**

**Monoclonal antibody subgroup**	**8/5**	**3/1**	**3**	**8/4**	**10/6**	**20/1**	**26/1**	**30/4**
Philadelphia	+	+	-	+	-	-	-	-
Allentown/France	+	+	-	-	-	-	-	+/−^*a*^
Benidorm	+	+	-	-	-	+	-	-
Knoxville	+	+	+	-	-	-	-	-
OLDA	+	-	-	+	-	+/−^*a*^	+	+/−^*a*^
Oxford	+	-	-	+	-	-	-	+/−^*a*^
Heysham	+	-	+	-	-	-	-	-
Camperdown	+	-	-	-	-	-	+	+
Bellingham	+	-	-	-	+	+	+	+

^*a*^ Variable results with different strains.

The structural characterization of LPS of *L. pneumophila* identified several specific chemical attributes which differs it from the LPS molecules of other Gram-negative bacteriareviewd in [17]. Particularly the *O*-antigen homopolymer structure consists of an unusual residue, 5-acetamidino-7-acetamido-8-*O*-acetyl-3, 5, 7, 9-tetradesoxy-*D*-glycero-*D*-galacto-nonulosonic acid (legionaminic acid) and its derivates [18-20].

A central step in understanding the correlation of the LPS structure and pathogenesis of *L. pneumophila* was the description of the genetic background of LPS molecules by Lüneberg and colleagues [21]. More precisely, a genetic locus composed of at least 28 open reading frames (ORF) is essential in LPS core oligosaccharide biosynthesis and LPS *O*-chain biosynthesis. The genes of this 31-36 kb cluster have characteristic functions required for the synthesis, transport, translocation and modification of LPS components. The *lag-1* gene of this biosynthesis locus encodes for an *O*-acetyltransferase which is responsible for the 8-*O*-acetylation of legionaminic acid [22]. Strains carrying a functional *lag-1* synthesize an LPS epitope that reacts with the mAb 3/1 (initially named mAb 2 [23]) of the Dresden monoclonal antibody panel. This epitope is assumed to contribute to an increased virulence [22,24] since mAb 3/1^+^ strains represent the most prominent subgroup of clinical *Legionella* isolates. In contrast, strains lacking *lag-1* carry mainly deacetylated LPS molecules. These mAb 3/1^-^ strains comprise only a small number of clinically identified *L. pneumophila* strains in immunocompetent patients [9,10].

Besides the mAb 3/1 specific *O*-acetylation of the legionaminic acid epitope, to date it remains elusive how strain specific mAb-reactivities can be explained. Increased understanding of the genetic background and structural LPS properties of the different Sg1 strains could help to comprehend subgroup distributions among clinical and environmental isolates [9,16,25-27] and would deliver more insight in the role of LPS in the *L. pneumophila* life cycle.

To achieve this goal, we analyzed the LPS-biosynthesis loci of at least one member of each mAb-subgroup (excluding mAb-subgroup Oxford) of the *L. pneumophila* Sg1. In this study we focused on the genetically composition of the loci and putative genotype-phenotype correlations according to the Dresden panel of mAbs.

## Results and discussion

### Two regions within the LPS-biosynthesis locus

To gain insight into the genetic composition and arrangement of the LPS biosynthesis locus we analyzed the loci of 14 *L. pneumophila* Sg1 strains. The strains represent members of all mAb–subgroups that can be distinguished by the Dresden monoclonal antibody panel (Table 1) besides the extremely rare mAb-subgroup Oxford. The LPS biosynthesis loci of five strains were newly sequenced for this study. These were: Camperdown 1 and Heysham 1 of the rarely found subgroups of the same name [9,25] and the strains Uppsala 3, Görlitz 6543 and L10/23. Eight LPS biosynthesis loci were obtained from complete genomes that have been published previously. Furthermore, for strain RC1 (mAb subgroup OLDA) the biosynthesis locus was available as well (Table 2).

**Table 2:** **LPS biosynthesis loci obtained from sequenced genomes of *L. pneumophila *Sg1 strains**

**Strain**	**mAb subgroup**	**Accession no.**	**Reference**
Alcoy 2300/99	Knoxville	GenBank: NC_014125.1	[28]
Corby	Knoxville	GenBank: NC_009494.2	[29]
L10/23 (Ulm)^*^	Knoxville	EMBL: HF545881	this study
Uppsala 3^*^	Knoxville	EMBL: HE980445	this study
Paris	Philadelphia	GenBank: NC_006368.1	[30]
Philadelphia 1	Philadelphia	GenBank: NC_002942.5	[31]
HL 0604 1035	Bellingham	EMBL: FQ958211	[32]
Görlitz 6543^*^	Bellingham	EMBL: HF678227	this study
Camperdown 1^*^	Camperdown	EMBL: HE980447	this study
Heysham 1^*^	Heysham	EMBL: HE980446	this study
130b (Wadsworth)	Benidorm	EMBL: FR687201	[33]
Lens	Benidorm	GenBank: NC_006369.1	[30]
Lorraine	Allentown	EMBL: FQ958210	[32]
RC1^*^	OLDA	EMBL: AJ277755	[21]

* only LPS biosynthesis locus sequenced.

The LPS-biosynthesis locus of each of the analyzed *L. pneumophila* Sg1 strains contained at least 28 ORFs and ranged in size from 30,644 bp (strain Lorraine) to 35,888 bp (strain 130b) with an average locus size of 33,398 bp respectively. The average ORF size within the locus was approximately 1 kb. The complete LPS-biosynthesis locus had a slightly lower % GC content (~ 35%) than the adjacent regions (~ 38%) and the total genome (~ 38.5%), respectively.

**Figure 1:**
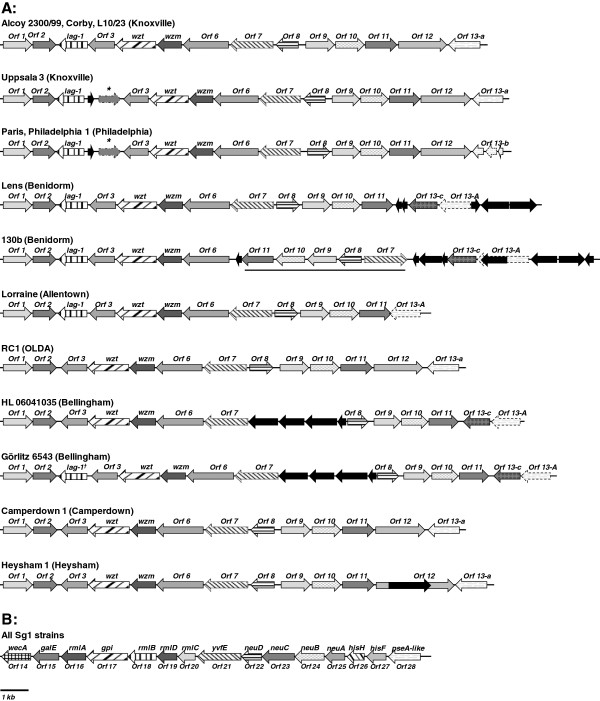
**Structural representation of the LPS-biosynthesis locus.** Shown are the LPS-biosynthesis loci of 14 *L. pneumophila* Sg1 strains and the corresponding monoclonal subgroup (in brackets). Strains Alcoy 2300/99, Corby and L10/23, and Paris and Philadelphia 1, respectively had the same genetic structure and monoclonal subtype and were therefore shown in one scheme. The numbering of ORFs was adopted by [21]. **A:** shows the Sg1-specific 18 kb region (ORFs 1-13) and **B:** shows the 15 kb region (ORFs 14-28). The direction of transcription is indicated by arrowheads. The filled black arrows indicate transposases/phage-related proteins. Grey shades and hatched patters serve to distinguish ORFs. Asterisk in Uppsala 3, Philadelphia 1 and Paris represents a partial ORF 2 duplication (ORF 2 like) as described by [46]. Underlined ORFs 7–11 in strain 130b represent an inversion. Görlitz 6543 carries a truncated *lag-1* marked with ^†^.

Structural and comparative analysis of the loci confirmed a highly conserved 15 kb region from *wecA* (ORF 14) to *lpg0748* (ORF 28) according to the Philadelphia genome as shown previously [34]. Additionally, all ORFs within this region were consistently orientated into the same direction (Figure 1A and B).

A second region within the locus of 18 kb in size is spanning from *lpg0779* (ORF 1) to *lpg0764* (ORF 13). Here, the structural composition and the orientation of ORFs as well as the total amount of putative ORFs was less conserved. Interestingly, many transposases and phage related genes were present in 8 strains (Figure 1A).

The heterogeneous nature of the 18 kb region and the extremely high conserved 15 kb region found in our study are largely in agreement with earlier results. These proposed to separate the locus into a Sg1 specific and a *L. pneumophila* specific region [34,35]. Microarray analysis of Sg1 and non-Sg1 strains have identified a 13 kb region (ORF 16–28) which is present in all *L. pneumophila* strains and a 20 kb region (ORF 1–15) generally found in all Sg1 strains [34]. The two regions were defined based on the LPS-biosynthesis loci of the Sg1 strain Paris [30].

To determine the putative breakpoint between both regions is difficult. However, based on our analysis of the structural composition we would rather separate the LPS biosynthesis locus between *lpg0763* (ORF 13) and *wecA* (ORF 14). This is in agreement with recent data, since the genes *wecA* (ORF 14) and *galE* (ORF 15) were demonstrated to be present in non-Sg1 strains with lower amino acid similarities when compared to Sg1 strains (55-61%) [35].

The initially mentioned ORF 13 is located next to the breakpoint region. In total, four different types of ORFs were found in the analyzed region of Sg1 strains here named ORF 13-a, -b, -c and –A. In each of the strains Lens, 130b, HL 06041035 and Görlitz 6543 two ORFs were found. These strains carried a putative conserved protein of unknown function (here referred to as ORF 13-A). A transposase-disrupted ORF 13-A was present in strain 130b (Figure 1A). Additionally, the strains carried an ORF which shared features of the radical S-adenosylmethionine (SAM) superfamily (CDD: cd01335) named ORF 13-c (Additional file 1: Table S2). Interestingly, all these strains lacked the ORF 12. However, even though the strain Lorraine lacked ORF 12 as well, it carried only a single ORF 13-A variant.

A distinct ORF of unknown function with amino acid similarity to ORF 13-A of only 38%, here named ORF 13-a, was present in the remaining strains with the exceptions of a truncated form in strains RC1, Philadelphia 1 and Paris. Philadelphia 1 and Paris shared high similarities with ORF 13-a but a deletion led to a frame shift resulting into three smaller fragments (pooled as ORF 13-b) (Table 3).

**Table 3:** **Amino acid similarity of the *L. pneumophila *Sg1 specific LPS-biosynthesis region from *lpg0769-lpg0761 *(ORF 1 - ORF 15) of strain Philadelphia 1 to other Sg1 strains**

Amino acid similarity [%]*														
**Gene name of *L. pneumophila***		**Philadelphia#**	**Knoxville#**				**Benidorm#**		**Bellingham#**		**Allentown#**	**OLDA#**	**Camperdown#**	**Heysham#**
**Sg1 strain Philadelphia 1**		**Paris**	**2300/99 Alcoy**	**Corby**	**Uppsala 3**	**Ulm**	**130b**	**Lens**	**HL 0604 1035**	**Görlitz 6543**	**Lorraine**	**RC1**	**Camperdown 1**	**Heysham1**
***lpg0761 (galE)***	ORF 15	100	100	100	100	100	97.1	96.0	99.8	99.8	99.8	100	100	100
***lpg0762 (wecA)***	ORF 14	100	99.5	99.5	99.5	99.5	93.4	93.1	93.7	93.7	93.4	98.8	99.5	99.5
***lpl0803***^**A**^	ORF 13	-	40.3	40.3	40.3	40.3	trans.^c^	100	98.2	98.2	96.6	41.8	40.3	40.3
***lpg0765***	ORF 12	100	98.6	98.7	98.6	98.6	-	-	-	-	-	98.7	98.6	trans.^c^
***lpg0766***	ORF 11	100	96.6	96.6	96.6	96.6	93.2	93.2	93.7	93.7	93.1	96.6	96.6	96.6
***lpg0767***	ORF 10	100	96.2	96.2	96.2	96.2	96.6	97.1	98.9	98.9	97	95.6	96.2	96.2
***lpg0768***	ORF 9	100	30.6	30.6	30.6	30.6	98.4	99	99	99	98.9	99.4	30.6	30.6
***lpg0769***	ORF 8	100	31	31	31	31	97.9	97.4	98.4	98.4	97.4	100	31	31
***lpg0770***	ORF 7	100	90.6	90.6	90.6	90.6	32	31.9	31.9	31.9	99.8	99.9	90.6	90.6
***lpg0771***	ORF 6	100	38.8	38.7	38.7	38.7	38.8	99.1	100	100	38.8	38.6	99.1	38.7
***lpg0772 (wzm)***	ORF 5	100	100	100	100	100	100	100	100	100	100	100	100	100
***lpg0773 (wzt)***	ORF 4	100	99	99.6	100	100	100	99.6	100	99.5	99	99.8	100	100
***lpg0774***	ORF 3	100	91.6	86.4	98.7	92.1	89	86.4	100	86.4	91.6	99.5	99.8	99.8
***lpg0775***^**a**^		100		-	100	-	-	-	-	-	-	-	-	-
***lpg0776***^**b**^		100	-	-	100	-	-	-	-	-	-	-	-	-
***lpg0777 (lag-1)***		100	96.8	94.9	100	96.8	94.9	94.9	-	94.7^†^	96.8	-	-	-
***lpg0778***	ORF 2	100	97.9	97.4	100	97.7	97.4	97.4	99.6	96.5	97.9	98.9	98.7	98.7
***lpg0779***	ORF 1	100	99.8	99.1	99.8	99.8	98.9	98.9	100	98.9	99.8	99.4	99.8	99.8

^#^ Monoclonal antibody subgroup according to the ‘Dresden’ panel.

* Determined by UPGMA clustering method based on multiple sequence alignment.

^A^ ORF 13 (lpg0764/lpg0764b/lpg0763) of Philadelphia 1 not displayed, ORF 13-A of strain Lens was used.

^a^ Partial duplication of ORF 2 (*lpg0778*).

^b, c^ Transposase; transposase disrupted.

^†^*Lag-1* of Görlitz 6543 has no functional start codon.

**Figure 2:**
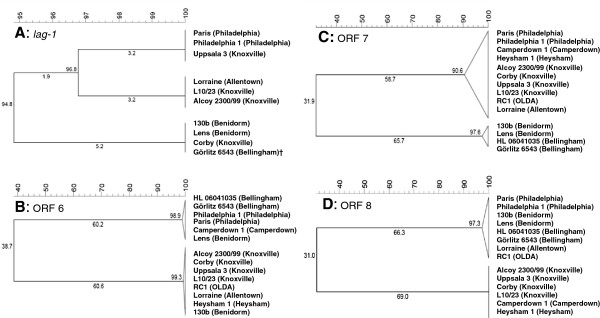
**Dendrogram of variable ORFs.** Multiple amino acid based cluster analysis using UPGMA (BioNumerics, Applied Maths NV, Belgium). The phylogenetic trees of gene *lag-1* and of the ORFs 6, 7 and 8 are shown. ORF 9 is identical to the phylogenetic tree of ORF 8 and is therefore not shown. Similarity values and branch distances were depicted in percentages [%]. The strain-specific mAb-subgroup is indicated in brackets. The mutated start codon of *lag-1* of Görlitz 6543 was neglected for similarity analysis and is indicated with ^†^.

Underlined numbers indicate different clusters of corresponding ORF (see also Figure 2).

The highly conserved 15 kb region (ORF14 – ORF 28) is not completely shown and only reflected by WecA and GalE.

### A conserved region found in all serogroup 1 strains

Within the conserved region several genes were found which are proposed to be involved in the biosynthesis of the highly acetylated core region which is composed of mannose, N-acetyl-glucosamine (GlcNAc), N-acetyl-quinovosamine (QuiNAc) and rhamnose residues [19]. A vast number of ORFs, more specifically ORF 21 through 25 and 28, were recently reported to facilitate the biosynthesis of the repetitive legionaminic acid residues of the *O*-antigen [18,36]. The pyrodoxal-phosphate dependent aminotransferase (ORF 21), the acetyltransferase *neuD* (ORF 22) and a dehydratase (*lpg0966*) located outside of the locus are likely to synthesize the precursor molecule of legionaminic acid, UDP-*N,N’*-diacetylbacillosamine (UDP-Bac2Ac4Ac) [37]. Contradictory to our findings, functions of the *neuD* products are described highlighting that the acetyltransferase is involved in Lag-1-independent *O*-acetylation of few legionaminic acid residues close to the LPS-core of *L. pneumophila*[21,38,39]. Furthermore, the adjacent genes encoding for NeuC (ORF 23), NeuB (ORF 24) and NeuA (ORF 25) were recently identified to be involved in the subsequent biosynthetic processes converting UDP-Bac2Ac4Ac into CMP-5,7-diacetamido-3,5,7,9-tetradeoxy-D-*glycero*-D-*talo*-nonulosonic acid (CMP-Leg5Ac7Ac) [36]. Moreover, the ORF 28 is homologous to the *ptmG* gene of *Campylobacter jejuni* (Cj1324) which converts the CMP-Leg5Ac7Ac residue to CMP-5-acetamidino-7-acetamido-3,5,7,9-tetradeoxy-D-*glycero*-D-*talo*-nonulosonic acid (CMP-Leg5Am7Ac) [40], the dominant residue of the *O*-antigen of non-Sg1 strains of *L. pneumophila*[41]. A functional correlation of the ORFs of this region is supported by recent transcriptomic data of strain Paris in which the ORFs 21-17 and 28-22 were transcribed as operons [42]. Since all analyzed Sg1 strains and a broad number of non-Sg1 strains carry ORF 28 [35,43,44] it can be assumed that CMP-Leg5Am7Ac is a common residue of the *L. pneumophila* LPS molecule which might subsequently become modified in a mAb-subgroup or even strain specific manner.

### Three clusters of the *O*-acetyltransferase Lag-1

A well examined phenotype variation is linked to the presence and absence of the *lag-1* gene. *Lag-1* encodes for an *O*-acetyltransferase that conferred reactivity with mAb 3/1 and is exclusively found in Sg1 strains. Our results revealed three clusters of the *lag-1* genes, although without any detectable relation to the mAb-subgroup switch which supports recent findings [45] (Figure 2A). *Lag-1* was previously reported to be involved in mAb-subgroup switches of different strains. However, this was generally due to gene deletion or loss-of-function mutations of *lag-1*[46-49]. Complete and functional *lag-1* genes were present in all mAb 3/1^+^ strains and were absent in all mAb 3/1^-^ strains. Besides that, the Philadelphia subgroup strains (Philadelphia 1 and Paris) as well as the Knoxville-subgroup strain Uppsala 3 carried a transposase and a partial duplication of ORF 2 adjacent to *lag-1*. Bernander et al. reported the region from ORF 2 to ORF 3 as unstable [46]. Looping out of the intermediate located *lag-1* gene is assumed to be a potential consequence. Under *in vitro* conditions the deletion of the *lag-1* gene occurred at with frequency of 10^-6^ to 10^-7^ (C. Lück, unpublished results). Detailed analysis of the region from ORF 2 to ORF 3 including *lag-1* of these strains revealed remarkably high similarities of Uppsala 3 to the Philadelphia-subgroup strains Philadelphia 1 and Paris (>98-100%) whereas the remaining Knoxville-subgroup strains clustered in a different group (Table 3; Figure 2A). The high similarity of this 4 kb region between strain Uppsala 3 and the strains Paris and Philadelphia 1 may indicate horizontal gene transfer of this region. However, this had no impact on the specific mAb reactivity for all other analyzed Knoxville-subgroup strains. Horizontal gene transfer between strain Paris and Philadelphia 1 was recently reported for a large genome fragment which also harbored the LPS biosynthesis locus [32]. These observations are confirmed by our results since the LPS biosynthesis loci of both strains are almost identical in composition, arrangement and amino acid similarity (Additional file 1: Table S2). A truncated *lag-1* gene was found in the strain Görlitz 6543 (mAb-subgroup Bellingham) as recently reported [49]. The whole gene is present but carries a mutated start codon. Since Görlitz 6543 showed no reactivity with mAb 3/1 it was assumed that the mutation significantly impairs the production of a functional *O*-acetyltransferase. Phylogenetic analysis showed 99.9% amino acid similarity of Görlitz 6543 to Corby (mAb-subgroup Knoxville), 130b and Lens (both mAb-subgroup Benidorm) (Figure 2A).

### ABC-transporter genes *wzt* and *wzm* as Sg1-specfic marker region

Noticeable conserved genes within the heterogenic region were *wzt* (ORF 4) and *wzm* (ORF 5) which are almost identical among all analyzed Sg1 strains (Figure 1A, Table 3). *Wzm* encodes for a protein containing a transmembrane domain while *wzt* encodes for a nucleotide binding domain of an ABC transporter system which mediates the *O*-antigen translocation across the inner membrane [50]. Recently, both genes were evaluated as marker genes for PCR based discrimination between *L. pneumophila* Sg1 and non-Sg1 strains [35]. The ABC transporter-dependent *O*-antigen pathway interacts with WecA (ORF 14), an UDP-GlcNAc-1-transferase which initiates *O*-chain biosynthesis at the cytoplasmic site of the inner membrane [50]. The low amino acid similarity of WecA between Sg1 and non-Sg1 that was described recently combined with the absence of *wzm* and *wzt* in non-Sg1 genomes [35] indicate a different *O*-chain biosynthesis mechanism for non-Sg1 strains than found in Sg1 strains.

### ORF 6 through 11 involved in *O*-antigen modification

The most variable region within the Sg1-specific region in terms of low similarities on the amino acid level and the diverse arrangement of single ORFs was found from ORF 6 to ORF 11. The strains of mAb-subgroup Benidorm 130b and Lens were almost identical regarding the amino acid similarities of the single ORFs within the Sg1-specific region. Interestingly, strain 130b carried a large inverted fragment containing ORF 7 to ORF 11 (Figure 1A). This region was surrounded by transposases suggesting their potential contribution to the inversion. Since the strain 130b showed no altered reactivity pattern using the Dresden panel compared to other Benidorm strains it could be stated that the inversion had no detectable effect on the LPS phenotype detected by monoclonal antibodies.

The adjacent ORF 6 showed a high degree of variability between *L. pneumophila* Sg1 strains represented by two clusters of low amino acid similarity (<39%) (Figure 2B). Interestingly, the two analyzed strains of the mAb-subgroup Benidorm, 130b and Lens, cluster into two distinct groups. This either indicates that the product of ORF 6 has probably no effect on the LPS structure of strains of the same monoclonal subgroup or that it has the same function despite low similarity.

However, ORF 6 products might be involved in the establishment of a mAb-subgroup discriminating epitope. More precisely, only the mAb-subgroups Heysham and Knoxville react with mAb 3. This indicates a similar epitope which in turn could possibly be traced back to specific ORFs within the Sg1-specific region. However, strains of both mAb-subgroups were highly homologous regarding the whole LPS-biosynthesis with the exception of lag-1 which is present in Knoxville strains. (Figure 2B, Table 3). In addition, the strain Camperdown 1, not reacting with mAb 3, carried a very similar LPS-biosynthesis locus as Heysham 1 and the Knoxville strains. However, it is the single ORF 6 in which Camperdown 1 clusters differently to Heysham 1. It can be assumed that the combination of ORF 6 to 9 which is exclusively found in Knoxville and Heysham strains leads to reactivity with mAb 3. Another ORF 6 as found in the genetically very similar strain Camperdown 1 could alter the LPS epitope and is thereby not recognized by mAb 3. Furthermore, the mAb 3 epitope was not influenced by *O*-acetylation of the legionaminic acid residue since the Knoxville strains were mAb 3/1^+^ and carried the *lag-1* gene whereas the strain Heysham 1 is negative for both markers.

### Modification of legionaminic acid in transposon mutants

Two additional ORFs, ORF 8 and ORF 9, within in the highly variable region from ORF 6 to ORF 11 are most likely involved in *O*-antigen modification. The genetic nature of the ORF 8 products displayed two different clusters which was comparable to the clustering of ORF 9. Both clusters share poor amino acid similarities of 31% (ORF 8) and 30.7% (ORF 9) (Table 3, Figure 2D). These differences in amino acid similarity were also reflected by the ORF orientation. Both ORFs were orientated into opposite directions in strains of the mAb-subgroups Knoxville, Camperdown and Heysham which form a separate cluster in both ORFs (Figure 1A). For the remaining mAb-subgroups (Philadelphia, Allentown, Benidorm, Bellingham and OLDA) the ORFs are oriented into identical directions. *In silico* analysis of these loci predicted a five-gene operon from ORF 8 to ORF 12 suggesting a coupled functional entity [51]. These strains were also grouped into a single cluster. However, recent transcriptomic data obtained from strain Paris revealed a four-gene operon which lacks ORF 8 [42]. For all strains regardless of the distance in the phylogenetic tree BLASTP predicted a methyltransferase function for ORF 8 [48,52] and a siliac acid synthetase function (neuB family) for ORF 9 [21].

It is reported that the putative methyltransferase encoded by ORF 8 is responsible for *N*-methylation of the 5-acetimydyol amino group of the legionaminic acid [48,52]. This is supported by studies on the legionaminic acid pathway of *Campylobacter*. The *ptmH* gene (Cj1325) of *C. jejuni* is a homologue of ORF 8 of the Knoxville, Camperdown and Heysham subgroup cluster (Figure 2D) [40]. The *ptmH* product catalyzes the modification of CMP-Leg5Am7Ac to the *N*-methylated residue CMP-5-acetimidoyl (*N*-methyl) amino-7-acetamido-3,5,7,9-tetradeoxynon-2-ulosonic acid (CMP-Leg5AmNMe7Ac), the main residue of the Sg1 *O*-antigen. Disruption of ORF 8 in the Bellingham-subgroup strain Görlitz 6543 led to loss-of-reactivity with the Bellingham-subgroup specific mAb 10/6 and mAb 20/1 and resulted in a mAb-subgroup switch from subgroup Bellingham to Camperdown. In similar mutants of the mAb 3/1^+^ strain 130b the reactivity with mAb 20/1 was also lost when ORF 8 or ORF 11 was disrupted leading to a switch from mAb-subgroup Benidorm to Allentown. The wild type strains 130b and these mutants did not react with mAb10/6. This supported the assumption that the mAb 3/1-specific epitope generated by the *O*-acetyltransferase Lag-1 masks the *N*-methyl group and hinders binding of mAb 10/6 [48]. This is in agreement with earlier observations which reported a correlation between ORF 8 and *N*-methylated legionaminic acid residues for the mAb 3/1^-^ strain RC1 [52]. However, the fact that mutants of both strains, 130b and Görlitz 6543, lost the reactivity with mAb 20/1, indicated that ORF 8 and/or ORF 11 are also involved in the generation or modification of another epitope which is not blocked by the *O*-acetyl group.

To find putative ORF candidates, next to ORF 8, that are responsible for synthesis or modification of the common epitope bound by mAb 20/1, we looked for similar but unique ORFs within the Sg1-specific region of Bellingham- and Benidorm-subgroup strains. Phylogenetic analyses identified ORF 7 as a putative subgroup discriminating gene since the mAb-subgroups Benidorm and Bellingham clustered in specific separate group when compared to the other mAb-subgroups (Figure 2C). The presence of two different ORF 7 variants is in agreement with recent results obtained by subgroup specific PCR amplification [49].

## Conclusions

Characterization of the LPS-biosynthesis loci of *L. pneumophila* Sg1 strains revealed two mayor regions: A Sg1-specific region of 18 kb and a conserved 15 kb region containing genes found in Sg1 and non-Sg1 strains. The conserved region carries genes involved in outer core and *O*-chain biosynthesis of LPS molecules.

The variable and heterogeneous Sg1-specific region raised questions concerning the genetic basis for subgroup specific mAb-reactivity. Switches from one monoclonal subtype to another in transposon induced mutants gave a first indication for the function of different gene products. The most variable region from ORF 6 to ORF 11 is likely involved in *O*-antigen modifications and could deliver more insight in the strain specific LPS structures and more important, in strain specific virulence. The ORFs within this region could act in a pathway-like manner explaining the broad variability of the LPS molecule among the Sg1 strains. Furthermore, it is also not excluded that each ORF of this region has an own function in the late modification of legionaminic acid derivates which could be regulated in a life cycle or growth phase-depended way. Further studies using specific mutation in these ORFs, mRNA assays and chemical analysis are required in order to elucidate the role of different genes in the synthesis of the subgroup specific structures in different strains.

## Methods

### Phenotypic and genotypic characterization of L. pneumophila strains

*Legionella pneumophila* Sg1 strains Camperdown 1 (ATCC 43113), Heysham 1 (ATCC 43107) [23], Uppsala 3 [46] and Görlitz 6543 [49] were grown on buffered charcoal yeast extract (BCYE) agar plates (Oxoid, Germany) for 48 hr at 37°C under a 5% CO_2_ atmosphere. Monoclonal subgrouping was accomplished using the Dresden panel of mAb as described elsewhere [13,16].

### DNA extraction and sequence generation

DNA was extracted using the EZ1 DNA Tissue Kit (Qiagen, Germany). Prior to sequencing DNA fragments of the LPS-biosynthesis locus were PCR-amplified using GoTaq polymerase (Promega, US-WI) and LPS-specific primers (Additional file 2: Table S1) which were designed based on published *L. pneumophila* genomes. Initial denaturation was carried out at 95°C for 2 min followed by 30–35 cycles: 95°C denaturation for 30 s, annealing at various temperatures for 1 min and elongation at 72°C for 1 min/kb. Final elongation for 5 min at 72°C completed the amplification protocol. The PCR result was checked on 1.5% agarose gel with 5 V/cm (LE Agarose, Biozym, Germany) and purified (MSB Spin PCRapace, Invitek, Germany) for sequence reaction.

Sequencing reactions were accomplished by a cycle-sequencing procedure on an automated DNA sequencing machine (ABI Prism 377, Applied Biosystems, US-CA).

The LPS-biosynthesis locus of the strain L10/23 was sequenced during a whole genome sequencing project. This strain was isolated during a cooling tower related outbreak in Ulm (Germany) in 2010 [53].

### Sequence annotation and analysis

Obtained sequences of Camperdown 1, Heysham 1, Uppsala 3, Görlitz 6543 and L10/23 were assembled using SeqMan (DNASTAR Lasergene 8, US-WI) and controlled against public databases using BLAST [54]. ORF annotation of all analyzed strains was accomplished with GeneMark.hmm [55] and Artemis [56]. The annotation and the numbering of ORFs was based on the initially described LPS-biosynthesis locus of strain RC1 (mAb-subgroup OLDA) [21] and if possible supplemented by further description of genes, gene products and their putative functions using BLAST, BLASTP [54,57], the LegionellaScope database of the MicroScope Microbial Genome Annotation Platform [58] and the Conserved Domain Database [59]. Since Lüneberg et al*.* analyzed the strain RC1 which had 30 ORFs the numbering of ORFs in other *L. pneumophila* Sg1 strains with deviating ORF numbers is not continual [21]. The genes *iraA* (ORF 29) and *iraB* (ORF 30) were not taken into account as part of the LPS-biosynthesis locus. Both formed a small 2-gene operon responsible for iron assimilation, infection and virulence [60].

The putative coding regions were compared to already known LPS-biosynthesis ORFs of published *L pneumophila* strains using the SeqMan program. The LPS-biosynthesis clusters of the strains were deposited in the EMBL database under the number [EMBL: HE980447] for strain Camperdown 1 (mAb-subgroup Camperdown), [EMBL: HE980446] for strain Heysham 1 (mAb-subgroup Heysham), [EMBL: HE980445] for strain Uppsala 3 (mAb-subgroup Knoxville), [EMBL: HF678227] for strain Görlitz 6543 (mAb-subgroup Bellingham) and [EMBL: HF545881] for strain L10/23 (mAb-subgroup Knoxville) (Table 2).

Sequence homologies of single ORFs were calculated based on multiple alignments using BioNumerics 6.0 (Applied Maths NV, Belgium) and BLASTP [57]. Cluster analysis was performed using the UPGMA method of the BioNumerics 6.0 software package.

The sequences of other LPS-biosynthesis loci were obtained from complete genomes of the following strains: Paris (mAb-subgroup Philadelphia) (GenBank: NC_006368.1), Lens (mAb-subgroup Benidorm) (GenBank: NC_006369.1), Philadelphia 1 (mAb-subgroup Philadelphia) (GenBank: NC_002942.5), Alcoy 2300/99 (mAb-subgroup Knoxville) (GenBank: NC_014125.1), Corby (mAb-subgroup Knoxville) (GenBank: NC_009494.2), Lorraine (mAb-subgroup Allentown) (EMBL: FQ958210), HL 06041035 (mAb-subgroup Bellingham) (EMBL: FQ958211), RC1 (mAb-subgroup OLDA) (EMBL: AJ277755) and 130b (mAb-subgroup Benidorm) (EMBL: FR687201.1) (Table 2) [21,28,29,31-34]. Since the genome of 130b is a draft version we closed a sequencing gap in scaffold 4 (position 918107 to 918206) using PCR and sequencing.

### Availability of supporting data

The data sets supporting the results of this article are available in the LabArchives repository, DOI:http://dx.doi.org/http://dx.doi.org/10.6070/H4WM1BBQ. It includes a list of all primers used for ORF amplification and sequence generation (Additional file 2: Table S1), a spreadsheet containing detailed information about the LPS-biosynthesis locus such as ORF identifier, ORF size and putative size of the translated ORF product (Additional file 1: Table S2) as well as the % GC content of the ORFs of the Sg1-specific region (Additional file 1: Table S3).

## Abbreviations

LPS: Lipopolysaccharide; mAb: Monoclonal antibody; ORF: Open reading frame; Sg1: Serogroup 1; GlcNAc: *N*-acetyl-glucosamine; QuiNAc: *N*-acetyl-quinovosamine; UDP-Bac2Ac4Ac: UDP-*N,N’*-diacetylbacillosamine; CMP-Leg5Ac7Ac: CMP-5,7-diacetamido-3,5,7,9-tetradeoxy-D-*glycero*-D-*talo*-nonulosonic acid; CMP-Leg5Am7Ac: CMP-5-acetamidino-7-acetamido-3,5,7,9-tetradeoxy-D-*glycero*-D-*talo*-nonulosonic acid; CMP-Leg5AmNMe7Ac: CMP-5-acetimidoyl (*N*-methyl) amino-7-acetamido-3,5,7,9-tetradeoxynon-2-ulosonic acid.

## Competing interests

The authors declare that they have no competing interests.

## Authors’ contributions

MP generated sequences of strains Camperdown 1 and Heysham 1, conducted comparative genetic and phylogenic studies, interpreted the results and drafted the manuscript. AT and SM generated sequences of strains Uppsala 3 and Görlitz 6543. KH generated the genome sequence of strain L10/23. JWM reviewed the manuscript. CL conceived and supervised the work, assisted with inspiring discussions and ideas, helped interpreting the results and reviewed the manuscript. All authors read and approved the manuscript.

## Supplementary Material

Additional file 2: Table S1 This document summarizes all primers used for amplification of LPS-biosynthesis ORFs and sequence generation. Click here for file

Additional file 1 **This file contains two spreadsheets containing the supporting data S2 and S3. Table S2.** Genes/proteins of the LPS-biosynthesis locus of *L. pneumophila* Sg1 strains. **Table S3.** Percentage GC-content of single ORFs, regions and the whole LPS-biosynthesis loci of *L. pneumophila* Sg1 strains. Click here for file
