# Supplementary material for: A structural comparison of lipopolysaccharide biosynthesis loci of Legionella pneumophila serogroup 1 strains
Source: BMC Microbiol. 2013 Sep 4;13:198. doi: 10.1186/1471-2180-13-198 (PMC3766260; doi:10.1186/1471-2180-13-198)
Supplement: Additional file 2: Table S1 — This document summarizes all primers used for amplification of LPS-biosynthesis ORFs and sequence generation. [file 1471-2180-13-198-S2.docx]

**Table S1: Primer used for amplification of LPS-biosynthesis ORFs and sequence generation**

| Name* | Sequence (5‘-3‘) | Tm [°C]^#^ |
| --- | --- | --- |
| Camp ORF 5mid | CGCCTATCAACGCTCTTGGA | 59.5 |
| Camp ORF 6mid | GCATAAGAACTTGGTTATAGCAGC | 59.5 |
| Camp ORF 7mid | CAGGAAACCCACTTGGGTGTA | 60 |
| Camp ORF 7up | CTGAAGATGTGGTTGAAAGAGAC | 58 |
| Camp ORF 7up2 | GCCGAGGGAGATTTATTTTTG | 59 |
| Camp ORF 9do | CCCATAGTATGTGATATGTCCTG | 60 |
| Camp ORF 9up | CAGGATCTTGGCATAGCTGAAC | 60.5 |
| Camp ORF 10do | CCGGTATGTACCACCAGCCT | 61.5 |
| Camp ORF 14do | GTAAGCGTGCTTTTTATGAGGGC | 60.5 |
| Camp ORF 15do | CATCTTGCGCGATAAGGTTTGC | 60.5 |
| Camp ORF 15mid | GAGGGCTTGTCTTTATTGGTCTT | 58 |
| Corby 7up | CAGAACTGCAGCATCTCCAC | 59.5 |
| Corby 7-9up | CTAGATCACATTCGTATCGTC | 57 |
| Corby 7-9do | GTTGACGAGATTGTATCTC | 50 |
| Corby 7-9do2 | GCGAATAACGTGACCATCTG | 57.5 |
| Hey ORF 6up | GACCTCACCCGGTTACTAATAAA | 59 |
| Hey ORF 6mid | CCTTAGTGGTACCGACTGC | 59 |
| Hey ORF 7up | TTTGGTCAAGAGTTTCCTGGC | 56.5 |
| Hey ORF 7mid | CGCCTCCTGCTAATACCTTC | 59.5 |
| Hey ORF 9up | TCTTTCCCCTTCTAGCTTTTGG | 58 |
| Hey ORF 9mid | CAGCAAAAAGTGCCGGATGTG | 60 |
| Hey ORF 9do | GCCGTACACCTTGTTGACGAG | 61.5 |
| Hey ORF 10do | CATGAGACCAAGGTTTAATAGCC | 59 |
| Hey ORF 11do | CTAGTTAAGGATTTAGTCGGGTG | 59 |
| Hey ORF 11do2 | CAAGGCTTGAGAAAATTATGGAGAC | 59.5 |
| Hey ORF 12do | GGGAGGGAATGTCGCAATAAAA | 58.5 |
| Hey ORF 12mid | GGGTAACACAATACAGCTGTTCT | 59 |
| Hey ORF 12up | CAACCCCACGATATCTACTACC | 60.5 |
| Hey ORF 13do | TCCTATGGAATAAAGATAAGAATTTTTTTG | 57 |
| lag Phil Au | CTGCACAGGGGGTAAGGAAAAATAAT | 61.5 |
| lag Phil Ku | GCAATAAGCAATAACAATGGCATA | 55.5 |
| lag Phil L | GTTAAGATAGAGTATAAATGGGGTGAG | 60.5 |
| lag Phil Ld | GTTAAGATAGAGTATAAATGGGGTGAG | 60.5 |
| lag phil Md | GCCAATTTGCACGCTACCATCC | 62 |
| lag Phil Mu | GCCAATTTGCACGCTACCATCC | 62 |
| lag Phil Ou | GTAATATTATTTCTGTTTCCTCATCATC | 58 |
| lag Phil Pd | GAAGGTGTAAGGGTAAAGTTGCAATAAG | 62 |
| lag Phil Rd | GCTTATATTTTTCCCCTGATTTTGACA | 59 |
| lag Phil Tu | CAACTCTAATAATAAGAGTGG | 52 |
| lag Phil MP1F | CGCTAAACTATTATCAGAAGAGGC | 59.5 |
| Lens LPS 6-8 do | GATACTAACAGCCAAGGTG | 54.5 |
| Lens LPS 7up1 | TCCAACCCAAGGAATTCCTG | 57 |
| Lens LPS 13do | GCAAGTCGTCTAGAAGTGGTCATC | 62.5 |
| Lens LPS 14do | GCTGGAGCAACATGAGATAATTGAG | 53 |
| LPS ORF 00up | CACAATATATAGCAAAAAGACTGC | 56 |
| LPS ORF 1do | CAAACCTTTAATCGCATCAATTTTAG | 57 |
| LPS ORF 2do | GAGTGGTAGCTGGAGAAGAATTTTC | 61.5 |
| LPS ORF 3 up | GCCGATCATACCGCACAAGTA | 60 |
| LPS ORF 3do | TCGACGATGAGGAAACAGAAATAAT | 58 |
| LPS ORF 4wzt up | GGGGGCGCTTAATCAGTTTG | 60 |
| LPS ORF 4wzt do | CCCCCTCATTTACAGCTCCATC | 62 |
| LPS ORF 4wzt do2 | GATTCACGGTTTCCATATCGTG | 58.5 |
| LPS ORF 5up | GATCTCAAAGGGCGTTACAGTCAAAC | 63 |
| LPS ORF 5do | GCAACAAGCCCCACAAGGAAAG | 62 |
| LPS ORF 6a up | GTAATAAATGAAGCTGGTGAGTTTG | 58 |
| LPS ORF 6b up | GAGGCAACAACAAACGGTCAGG | 60 |
| LPS ORF 6a do | CGCAAAAGGTATGATTAATGTAGGAG | 60 |
| LPS ORF 6b do | GCACTAGCGCATTCTTGTTGGTTC | 60 |
| LPS ORF 7do | CGCCATAAAGACCTCCCATCAAT | 60.5 |
| LPS ORF 8up | GACGGCTGGAGCAAAGAACCTAA | 62.5 |
| LPS ORF 8do | CCAATTTTTACAACGACCCTCAAG | 59.5 |
| LPS ORF 9up | GCCAATTTACCCTTCATAGTTTCG | 59.5 |
| LPS ORF 9do | GTAATTCCCAGCCATTTACCAGATC | 61.5 |
| LPS ORF 10up | GAGAAGATGGGAGGGTTTTTATTG | 59.5 |
| LPS ORF 10up2 | CGGAACGAACATCACAGCT | 57 |
| LPS ORF 10dorc3 | CCTTGGCCTCATGTTTATTCATGC | 61 |
| LPS ORF 10dorc5 | GGCTGGTGGCACATACCA | 58 |
| LPS ORF 11up | GTTCTTGTTACTGCACGGGATGTTG | 63 |
| LPS ORF 11up2 | GCATAGATCATCACGCTGCAG | 60 |
| LPS ORF 11do | GGATGGGGGCGATAAAGAATAAC | 60.5 |
| LPS ORF 11do2 | GCAAGCTTGCAACCATTGTGA | 58 |
| LPS ORF 12up | CGACAAATAAACAATGGCAATGGTG | 60.5 |
| LPS ORF 12do | CGGTTCAGCGCAAAGTGTTCC | 60.5 |
| LPS ORF 12do2 | GCTGCATAATCCTCCAGTAG | 57.5 |
| LPS ORF 13up | CTTTATTTCTTATTGTTGGTGGTCTGTC | 60.5 |
| LPS ORF 13do | CTGTGGCAAAAGCATGAGGGTAG | 62.5 |
| LPS ORF 13do2 | GTATCGTGATTCTGCATAAG | 53 |
| LPS ORF 14up1 | GCCTTGTTTGCGCAGCATAC | 57 |
| LPS ORF 14do | CCAATTGCTTTAATCTCTGG | 53 |
| LPS ORF 14do2 | GGAGGCGCTCCCTACATCTCC | 63.5 |
| LPS ORF 15up | CCGGCGCGACAGGATTTATAGGACG | 68 |
| LPS ORF 15up2 | GACTCAACCTGAAGATCCCTATG | 60.5 |
| LPS ORF 16up | GCTGGAAATAACTGACATCAATC | 57 |
| LPS ORF 16up2 | GATAATATCCACCAGGGCAGAG | 60 |
| LPS ORF 16up3 | GTACAAGACTGTATCCTCTGAC | 58.5 |
| LPS ORF 17up | GCAATATGCCAATGAATCATCTC | 57 |
| LPS ORF 17up2 | CGCAGTTAATTTAATTACTGC | 52 |
| LPS ORF 17do | GCTTATCGATTTGGCTAATG | 53 |
| LPS ORF 18up | GCAATTGATACCATTATTC | 49 |
| LPS ORF 18do | GAGTGAAATGGCAGAATG | 51.5 |
| LPS ORF 19up | GAAGGCCTACTTCAGCTCGCG | 63.5 |
| LPS ORF 19do | GCAAGTTGGTACCGAGATTATC | 58.5 |
| LPS ORF 20do | GTTATTAGGAATAACTGATCG | 52 |
| LPS ORF 21up | GATACAGTACTCGTTGGTTATCG | 59 |
| LPS ORF 22-a up | GTTGTTTTGCCTGGTGTGACTAT | 59 |
| LPS ORF 22-a do | GATATGAGGCGAGGAAAGCAG | 60 |
| LPS ORF 23up | GTGAAAGGTGTTATCAATTG | 51 |
| LPS ORF 23do | CAGAGGCATCCAAACAACG | 56.5 |
| LPS ORF 24up | GTACAATCCATTAGAGACGC | 55.5 |
| LPS ORF 24do | CAGTATGAGATGCTCAAGGC | 57.5 |
| LPS ORF 26up | GAATATGTCTTGGCATGCAG | 55 |
| LPS ORF 27up | CTAATATCTGATATTGCCAG | 51 |
| LPS ORF 27up2 | GATCAGGAAGGTACGCGTAAG | 60 |
| LPS ORF 28up | CATTGCTTCGCAAATGTTCGAG | 58.5 |
| LPS ORF 28do | CAATCCTGGATACATGCCG | 56.5 |
| LPS ORF 29up | CTTGAAGGCCATGTATAAC | 52.4 |
| LPS ORF 29do | GAATTGGCACTGTTTGGAATAG | 56.5 |
| LPS ORF 30up | GGCCGTTCATGCTGAGGAGGT | 63.5 |
| LPS ORF 30mid | GCGCAGGATGTGCTATAGGAA | 60 |
| LPS ORF 30do | CAAGCCACAGGCTACCTGATGC | 64 |
| NeuA-up | CCGTTCAATATGGGGCTTCAG | 60 |
| NeuA-do | CGATGTCGATGGATTCACTAATAC | 59.5 |

* For several primes a reverse complement primer was used

^#^ Tm [°C] = 69.3 + 0.41 (% G+C) – (650/primer length)
